# Predictors of the Extent of Carotid Atherosclerosis in Patients Treated with Radiotherapy for Nasopharyngeal Carcinoma

**DOI:** 10.1371/journal.pone.0116284

**Published:** 2014-12-31

**Authors:** Chuang Yuan, Vincent W. C. Wu, Shea Ping Yip, Dora L. W. Kwong, Michael Ying

**Affiliations:** 1 Department of Health Technology and Informatics, The Hong Kong Polytechnic University, Hung Hom, Kowloon, Hong Kong SAR, China; 2 Department of Clinical Oncology, The University of Hong Kong, Pokfulam, Hong Kong SAR, China; Katholieke Universiteit Leuven, Belgium

## Abstract

**Objective:**

To determine the predictors of the extent of carotid atherosclerosis in patients treated with radiotherapy (RT) for nasopharyngeal carcinoma (NPC).

**Methods:**

The present study investigated 129 post-RT NPC patients. Carotid atherosclerotic parameters, such as carotid intima-media thickness, carotid arterial stiffness and carotid plaque burden (plaque score, the presence of plaque and ≥50% stenosis) were assessed using ultrasonography. The association between carotid atherosclerotic parameters and nine potential predictors, including age, gender, post-RT duration, radiation dose, chemotherapy, diabetes mellitus, hypertension, hypercholesterolemia, and smoking, were determined using multiple regression. The cutoff values of age, post-RT duration and number of cardiovascular risk factors for the presence of carotid plaque or ≥50% carotid stenosis were analyzed using receiver operating characteristic (ROC) curve analysis. Multiple testing was corrected using Benjamini-Hochberg false discovery rate.

**Results:**

Age, post-RT duration and number of cardiovascular risk factors were significantly associated with carotid plaque burden (corrected *P* value, *P_cor_*<0.05). Age of 44.5 years (sensitivity = 99.2% and specificity = 50%, *P_cor_*<0.01) and post-RT duration of 8.5 years (sensitivity = 75.7% and specificity = 64.3%, *P_cor_*<0.001) were the cutoff values for detecting carotid plaque, while post-RT duration of 13.5 years (sensitivity = 66.7% and specificity = 71.6%, *P_cor_*<0.001) and 1.5 cardiovascular risk factors (sensitivity = 40.7% and specificity = 84.3%, *P_cor_*<0.05) were the cutoff values for screening ≥50% carotid stenosis.

**Conclusions:**

Age, post-RT duration and number of cardiovascular risk factors are significant predictors of carotid atherosclerosis in post-RT NPC patients. Post-RT NPC patients, who are at least 45 years old, with post-RT duration of 9 years or above, and/or have ≥2 cardiovascular risk factors, are more susceptible to carotid atherosclerosis.

## Introduction

Nasopharyngeal carcinoma (NPC) is common head and neck cancer in Southern China and Southeast Asia [1]. Since NPC is highly sensitive to radiation, and cervical lymph node metastasis is common in NPC patients (60–90%) [2], neck radiotherapy (RT) is a routine for treating or preventing the nodal metastasis in NPC patients. However, the ionizing radiation from RT damages the carotid artery and may lead to the development of carotid atherosclerosis.

It has been shown that post-RT NPC patients tend to have thicker carotid intima-media, higher prevalence of carotid plaque and/or greater degree of carotid stenosis [3]–[6], which result in higher incidence of cerebrovascular events [6]. Although evidences have demonstrated the high severity of radiation-induced carotid atherosclerosis, assessment of carotid atherosclerosis is not yet a routine practice in follow-up of patients treated with RT for NPC. Early diagnosis and prompt treatment of carotid atherosclerosis are important for preventing cerebrovascular events. Thus, it is necessary to identify the post-RT NPC patients who have high risk of carotid atherosclerosis so that early diagnosis can be made and prompt treatment can be given to the patients.

Although different predictors of carotid intima-media thickness (CIMT) and carotid stenosis in patients treated with neck RT have been reported in previous studies [7], [8], no previous studies have investigated the cumulative effect of conventional cardiovascular risk factors, such as diabetes mellitus, hypertension, hyperlipidemia and smoking, on radiation-induced carotid atherosclerosis in post-RT NPC patients. In addition, significant carotid stenosis (≥50% stenosis) is an important indicator of carotid atherosclerosis and is useful for predicting cerebrovascular events [9], [10]. However, the risk factors that help to predict ≥50% carotid stenosis in post-RT NPC patients are still unknown. Thus, the cumulative effect of cardiovascular risk factors on carotid atherosclerosis and the predictors of ≥50% carotid stenosis in post-RT NPC patients needs to be investigated.

The present study aimed to identify the predictors that were useful to predict the occurrence and severity of carotid atherosclerosis in post-RT NPC patients. The findings will help identifying patients who have high risk of carotid atherosclerosis after RT, and this facilitates early diagnosis and prompt treatment of carotid atherosclerosis in post-RT NPC patients.

## Methods

### Subjects

Post-RT NPC patients were recruited from the Department of Clinical Oncology of Queen Mary Hospital. The patients were invited to join the present study when they had the routine follow-up for NPC in the hospital. For subject recruitiment, the inclusion criteria were Chinese NPC patients older than 18 years and having completed RT for at least four years, while the exclusion criteria were more than one course of RT, history of carotid atherosclerosis prior to RT, previous carotid endarterectomy and carotid stenting.

This study was approved by the Human Subject Ethics Subcommittee of the Hong Kong Polytechnic University and the Institutional Review Board of the University of Hong Kong/Hospital Authority Hong Kong West Cluster. Written consent was obtained from all participated patients before the commencement of the interview and ultrasound examination.

### Clinical information

The information of age, gender, post-RT duration, radiation dose, chemotherapy, and history of carotid atherosclerosis and cardiovascular risk factors was obtained from archived clinical records. Individual face-to-face interviews in the patients were also conducted. The presence of cardiovascular risk factors was identified as follows: 1) DM, diagnosed with DM in the clinical record, taking medications to lower the glycemic level and/or fasting plasma (blood) glucose ≥7.0 (6.1) mmol/L [11]; 2) hypertension, diagnosed with hypertension in the clinical record, undergoing anti-hypertensive medications and/or the measured blood pressure ≥140/90 mmHg [12]; 3) hypercholesterolemia, diagnosed with hypercholesterolemia in the clinical record, undergoing medications to lower the cholesterol level and/or fasting total cholesterol ≥5.2 mmol/L [13]; 4) smoking, current smoker consuming 10 cigarettes per day for at least six months [8].

### Ultrasound examinations

The brachial blood pressure was measured using a sphygmomanometer (Tensoval; Hartmann, Germany). All ultrasound examinations were performed with the Esaote MyLab Twice ultrasound unit in conjunction with a 4–13 MHz linear transducer (Esaote, Genoa, Italy). The blood pressure measurements and ultrasound examinations were conducted in a 22°C air-conditioned examination room. For each subject, the brachial blood pressure was measured with the sphygmomanometer at the left upper arm in a sitting posture after rest for at least 10 minutes. The measured systolic and diastolic pressures were then inputted into the ultrasound unit for the evaluation of CAS. Subjects were then asked to lie supine on the examination couch with the neck slightly extended and the head turned away from the side under examination. Both the left and right carotid arteries of the subject were assessed.

**Figure 1:**
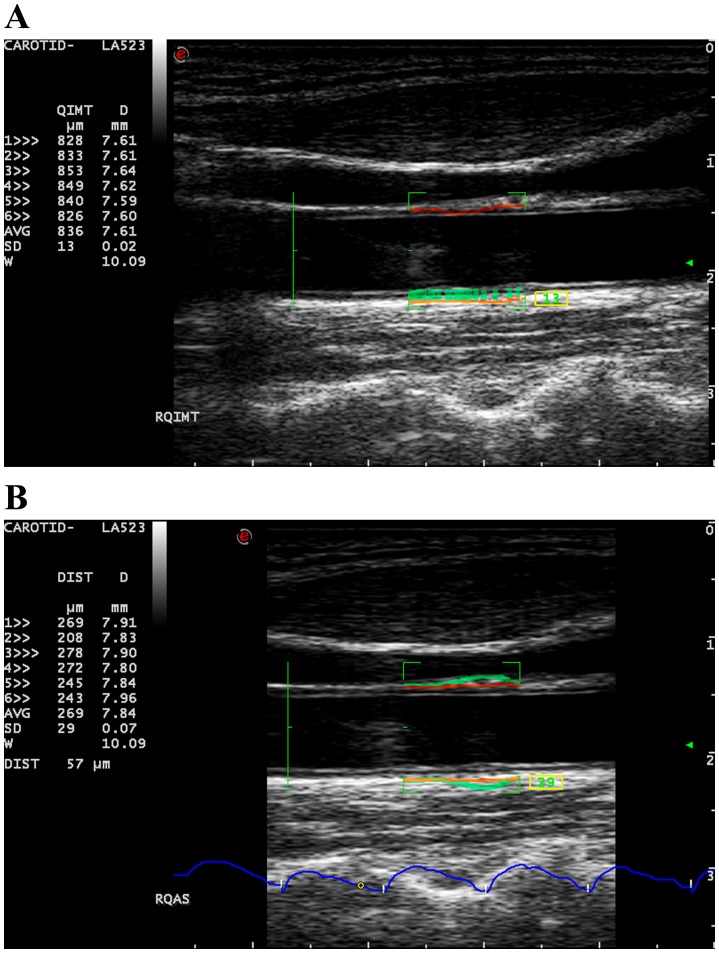
Measurements of carotid intima-media thickness and carotid arterial stiffness. **A**). Carotid intima-media thickness is assessed at 1-cm segment 1 cm proximal to the inferior end of carotid bulb using Radiofrequency-based Quality Intima-Media Thickness. **B**). Carotid arterial stiffness is measured at the same segment using Radiofrequency-based Quality Arterial Stiffness.

The automated quantification programmes of the ultrasound unit, RF-QIMT and RF-QAS, were used for evaluating CIMT and CAS respectively (Fig. 1). CIMT and CAS were measured at the same segment of the common carotid artery (CCA): 1-cm segment 1 cm proximal to the inferior end of carotid bulb. CIMT was evaluated at the far wall of the CCA while CAS was measured over the near and far walls. In the evaluation of CIMT and CAS, the mean and standard deviation (SD) of the measurements in six consecutive cardiac cycles were automatically and continuously recorded by the system, and the mean measurement with an SD of <20 µm for CIMT or <30 µm for stroke change in vessel diameter for CAS was obtained for data analysis. Each carotid artery was scanned three times for measuring CIMT and CAS.

In the evaluation of CAS, five arterial stiffness parameters, including distensibility coefficient (DC), compliance coefficient (CC), the indices of α and β, and pulse wave velocity (PWV), were investigated in the study. The lower DC and CC and the higher α, β and PWV suggest the stiffer the carotid artery. Detailed information of the equations of these parameters has been reported in our previous study [14].

**Figure 2:**
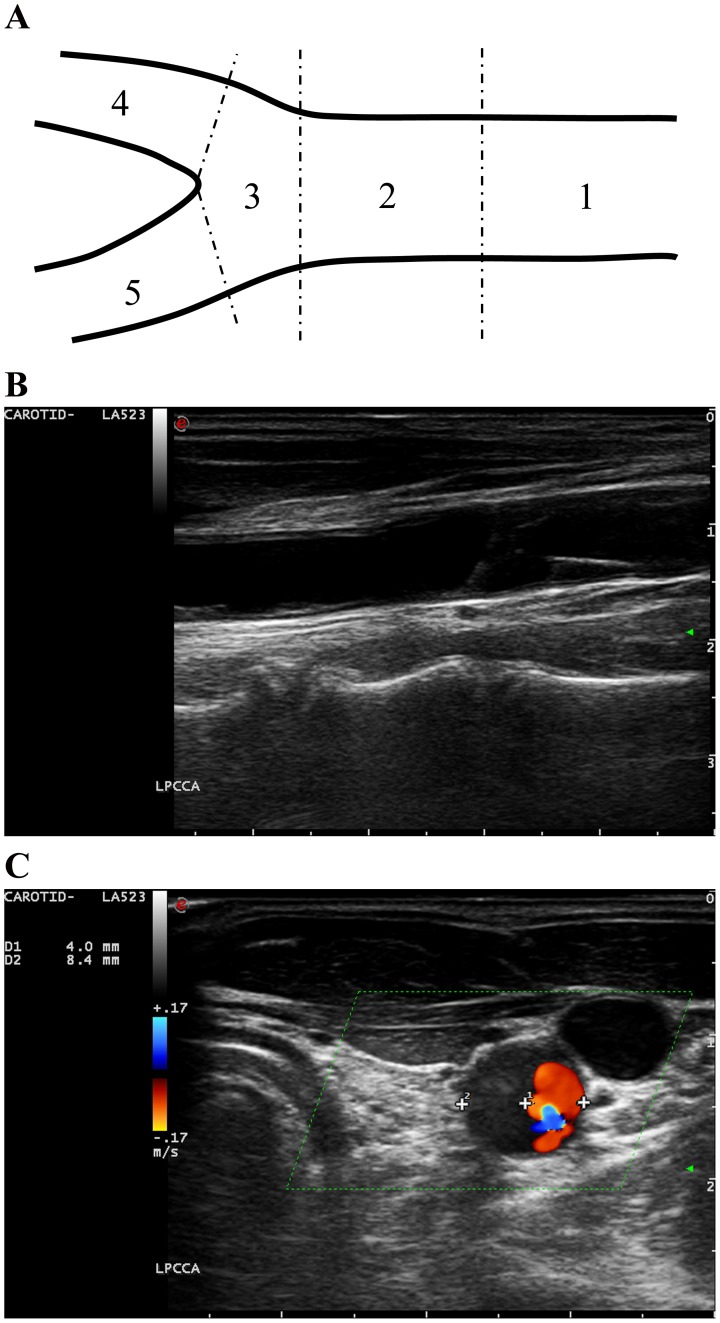
Assessment of carotid plaque burden. **A**). Five segments of the extra-cranial carotid artery: 1. proximal common carotid artery (≥2 cm proximal to carotid bifurcation); 2. distal common carotid artery (<2 cm proximal to carotid bifurcation); 3. carotid bulb and bifurcation; 4. internal carotid artery; and 5. external carotid artery. **B**). A longitudinal gray-scale image of a carotid plaque in the proximal common carotid artery. **C**). A transverse duplex ultrasound (gray-scale+Doppler) image of the carotid plaque in B. The plaque is echolucent with a unclear boundary between the lumen and the plaque. Therefore, Doppler ultrasound is applied to demonstrate the lumen at the stenosis. The degree of the carotid stenosis is expressed as the percentage reduction of lumen diameter (1-D1/D2 = 52.4%). Carotid plaque score is the summation of the degree of carotid stenosis of the five segments in both carotid arteries.

Using ultrasound, the carotid plaque burden of each subject was assessed. Any focal thickening >50% of the adjacent intima-media layer was identified as a carotid plaque [15]. Once a carotid plaque was identified, transverse gray-scale images of the plaque were obtained and the degree of carotid stenosis was expressed as a percentage reduction of lumen diameter at the most stenotic site (Fig. 2). Carotid plaque score was evaluated using an adjusted plaque scoring system [8]. In the scoring system, the carotid artery was divided into five segments: 1. proximal common carotid artery (≥2 cm proximal to carotid bifurcation); 2. distal common carotid artery (<2 cm proximal to carotid bifurcation); 3. carotid bulb and bifurcation; 4. internal carotid artery; and 5. external carotid artery. The degree of carotid stenosis in each segment was measured and carotid plaque score was expressed as the summation of the degree of carotid stenosis of all segments in both carotid arteries (Fig. 2).

### Statistical analysis

CIMT, CAS and carotid plaque score were continuous parameters, while the presence of carotid plaque and the presence of ≥50% carotid stenosis were categorical data. Continuous data were expressed as means ± SD. The normality of distribution was checked using Shapiro-Wilk test. Non-normally distributed parameters were transformed logarithmically. ANCOVA or logistic regression was used for the adjusted comparisons between study groups. Multiple regression was utilized for detecting the association between the extent of carotid atherosclerosis and nine potential predictors, including age, gender, post-RT duration, radiation dose, chemotherapy, DM, hypertension, hypercholesterolemia and smoking. The cutoff values of age, post-RT duration and the number of cardiovascular risk factors for the presence of carotid plaque or the presence of ≥50% carotid stenosis were analyzed using receiver operating characteristic (ROC) curve analysis. Multiple testing in each atherosclerotic parameter was corrected using Benjamini-Hochberg step-up false discovery rate controlling procedure [16]. All statistical analyses were performed using SPSS 20 (IBM, Armonk, New York, United States) and corrected *P* value (*P_cor_*)<0.05 was considered to be significant.

## Results

### Demographic information

In total, 129 post-RT NPC patients were included in the present study. The patients included 87 males and 42 females with a mean age of 55.3±8.8 years (range: 33 to 88 years). Conventional 2D RT of the neck was given to all patients. The mean radiation dose of the patients was 66.83±3.24 Gy (range: 58.00 to 73.72 Gy). There were 66 patients treated with RT alone and the remaining 63 patients were treated with chemo-radiotherapy. The mean post-RT duration of the patients was 12.5±6.0 years with a range of 4 to 37 years. The most common cardiovascular risk factor was hypercholesterolemia (n = 40), followed by hypertension (n = 35) and then by DM (n = 14). Only 5 patients were current smoker.

### Carotid intima-media thickness

In the prediction model using multiple linear regression, advancing age (*P_cor_*<0.01, standardized coefficients = 0.259) and male sex (*P_cor_*<0.01, standardized coefficients = 0.248) were significantly associated with higher CIMT in post-RT NPC patients (Table 1). None of the other parameters, such as cardiovascular risk factors, post-RT duration, radiation dose and chemotherapy, showed significant association with CIMT independently. Another regression model substituting the four cardiovascular risk factors with the number of these risk factors was used for assessing the cumulative effect of cardiovascular risk factors on CIMT. The result showed that the number of these risk factors was not associated with CIMT (Table 2).

**Table 1:** Corrected *P* values in multiple regression models for assessing the association between the ten potential predictors and carotid atherosclerotic parameters.

Parameters	Age	Gender	Post-RT duration	Radiation Dose	Chemotherapy	DM	Hypertension	Hypercholesterolemia	Smoking
CIMT	0.007**	0.007**	0.937	0.383	0.951	0.719	0.502	0.118	0.347
DC	<0.001***	0.736	0.003**	0.835	0.797	0.491	0.007**	0.054	0.084
CC	<0.001***	0.102	0.001**	0.921	0.672	0.449	0.005**	0.68	0.95
α	<0.001***	0.820	0.003**	0.981	0.276	0.821	0.017*	0.102	0.101
β	<0.001***	0.861	0.003**	0.997	0.292	0.810	0.016*	0.106	0.103
PWV	<0.001***	0.722	0.001**	0.867	0.667	0.339	0.001**	0.074	0.131
Carotid Plaque Score	0.049*	0.218	<0.001***	0.513	0.123	0.304	0.183	0.593	0.723
Presence of Carotid Plaque	0.015*	0.372	0.010*	0.485	0.447	0.922	0.755	0.700	0.712
≥50% Carotid Stenosis,	0.457	0.505	0.019*	0.994	0.273	0.178	0.380	0.609	0.063

*P_cor_<0.05,

**P_cor_<0.01,

***P_cor_<0.001.

*P_cor_* is corrected *P* value after false discovery rate correction for multiple testing. DM, diabetes mellitus; CIMT, carotid intima-media thickness; DC, distensibility coefficient; CC, compliance coefficient; PWV, pulse wave velocity.

**Table 2:** Ultrasonographic characteristics in post-RT NPC patients with different number of cardiovascular risk factors.

Parameters	Total n = 129	Groups with different number of cardiovascular risk factors			*P_cor_* a	*P_cor_* b
		0 n = 69	1 n = 33	≥2 n = 27		
Age, years	55.3±8.8	52.5±8.4	57.6±9.9	59.5±5.7	-	-
Gender (female/male), n	42/87	28/41	8/25	6/21	-	-
Chemotherapy, n (%)	63 (48.8%)	34 (49.3%)	16 (48.5%)	13 (48.1%)	-	-
Radiation Dose, Gy	66.83±3.24	66.87±3.45	67.02±3.02	66.48±3.00	-	-
Post-RT duration, years	12.5±6.0	12.7±6.3	11.8±5.7	13.2±5.9	-	-
CIMT, µm	720.40±164.13	681.72±132.23	765.73±162.17	763.84±214.43	0.956	0.984
DC, 1/KPa	0.013±0.008	0.015±0.009	0.011±0.006	0.012±0.008	0.706	0.706
CC, mm^2^/KPa	0.599±0.339	0.663±0.384	0.539±0.252	0.508±0.279	0.617	0.617
α	8.678±6.209	7.755±5.780	9.061±5.557	10.568±7.635	0.630	0.630
β	17.513±12.345	15.634±11.360	18.295±11.163	21.358±15.293	0.613	0.613
PWV, m/s	10.111±3.602	9.443±3.526	10.504±3.018	11.340±4.146	0.525	0.525
Carotid Plaque Score	1.41±1.36	1.19±1.18	1.17±0.90	2.25±1.89	0.030*	0.024*
Presence of Carotid Plaque n (%)	115 (89.1%)	60 (87.0%)	30 (90.9%)	25 (92.6%)	0.564	0.564
≥50% Carotid Stenosis, n (%)	27 (20.9%)	11 (15.9%)	5 (15.2%)	11 (40.7%)	0.028*	0.026*

*P_cor_<0.05.

a Association between ultrasonographic parameters and the number of cardiovascular risk factors.

b Comparisons between groups with ≥2 or <2 cardiovascular risk factors.

Age, gender, radiation dose, post-RT duration and chemotherapy were adjusted in all analyses. *P_cor_* is corrected *P* value after false discovery rate correction for multiple testing. CIMT, carotid intima-media thickness; DC, distensibility coefficient; CC, compliance coefficient; PWV, pulse wave velocity.

### Carotid arterial stiffness

With CAS as the phenotype (DC, CC, α, β and PWV), advancing age (*P_cor_*<0.001, standardized coefficients = −0.532, −0.434, 0.493, 0.489 and 0.474, respectively), longer post-RT duration (*P_cor_*<0.01, standardized coefficients = −0.76, −0.300, 0.276, 0.279 and 0.309, respectively) and the presence of hypertension (*P_cor_*<0.05, standardized coefficients = −0.208, −0.223, 0.185, 0.187 and 0.265, respectively) were significantly associated with lower DC and CC and higher α, β and PWV (i.e. stiffer artery) in post-RT NPC patients (multiple linear regression, Table 1). Other cardiovascular risk factors, radiation dose and chemotherapy did not show significant association with the five CAS parameters. In another regression model substituting the four cardiovascular risk factors with the number of these risk factors, results showed that there was no significant association between the number of the risk factors and CAS (Table 2)

### Carotid plaque score

Among the nine potential predictors, age and post-RT duration showed significant association with carotid plaque score (*P_cor_*<0.05, standardized coefficients = 0.184; and *P_cor_*<0.001, standardized coefficients = 0.349 respectively, Table 1). Cardiovascular risk factors and other potential predictors were not independently associated with the plaque score. In the regression model using the number of cardiovascular risk factors, the number of risk factors was positively associated with carotid plaque score (*P_cor_*<0.05, standardized coefficients = 0.185, Table 2). After the adjustment of age, gender, radiation dose, post-RT duration, and chemotherapy using ANCOVA, patients with two or more cardiovascular risk factors had significantly higher carotid plaque score than those with one or without cardiovascular risk factor (*P_cor_*<0.05, Table 2).

### The presence of carotid plaque

In the 129 post-RT NPC patients, carotid plaque was detected in 115 patients (89.1%). Advancing age (*P_cor_*<0.05, OR = 1.128, 95%CI: 1.024–1.244) and longer post-RT duration (*P_cor_*<0.05, OR = 1.347, 95%CI: 1.074–1.691) were significantly associated with higher prevalence of carotid plaque (multiple logistic regression, Table 1). However, other parameters did not have significant association with the presence of carotid plaque. In another regression model substituting the four cardiovascular risk factors with the number of these risk factors, results showed that no significant association was found between the number of cardiovascular risk factors and the presence of carotid plaque (Table 2).

Since age and post-RT duration were significantly associated with the presence of carotid plaque, ROC curve analysis was conducted to determine the cutoff values. Results showed that the cutoff value of age was 44.5 years (*P_cor_*<0.01, AUC = 0.757, sensitivity = 0.992 and specificity = 0.500) and that of post-RT duration was 8.5 years (*P_cor_*<0.001, AUC = 0.772, sensitivity = 0.757 and specificity = 0.643) for detecting carotid plaque. Carotid plaque was observed in 93.8% of patients aged 45 years or above, 94.6% of patients with a post-RT interval of at least 9 years, and 97.6% of patients with both at least 45 years old and a post-RT interval of at least 9 years. On the contrary, carotid plaque was only found in 56.3% of patients aged younger than 45 years, 75.7% of patients with a post-RT interval of less than 9 years, and 42.9% patients aged younger than 45 years and with a post-RT interval of less than 9 years.

### ≥50% carotid stenosis

In the present study, ≥50% carotid stenosis was found in 27 post-RT NPC patients (20.9%). Among the nine potential predictors, only post-RT duration (*P_cor_*<0.05, OR = 1.124, 95%CI: 1.026–1.232) was significantly associated with the presence of ≥50% carotid stenosis (multiple logistic regression, Table 1). Although independent cardiovascular risk factor did not show significant association with the presence of ≥50% carotid stenosis, increased number of cardiovascular risk factors was significantly associated with higher prevalence of ≥50% carotid stenosis when adjusted with age, gender, radiation dose, post-RT duration and chemotherapy (*P_cor_*<0.05, OR = 1.730, 95%CI: 1.062–2.817, Table 2).

Since post-RT duration and the number of cardiovascular risk factors were significantly associated with the presence of ≥50% carotid stenosis, ROC curve analysis was conducted to determine the cutoff values. Results showed that the cutoff values of post-RT duration and number of cardiovascular risk factors were 13.5 years and 1.5 for the presence of ≥50% carotid stenosis respectively (*P_cor_*<0.001, AUC = 0.709, sensitivity = 0.667 and specificity = 0.716; and *P_cor_*<0.05, AUC = 0.623, sensitivity = 0.407 and specificity = 0.843,). The presence of ≥50% stenosis was found in 38.3% of patients with post-RT duration of at least 14 years, 40.7% of patients with ≥2 cardiovascular risk factors, and 63.6% of patients with both post-RT duration of at least 14 years and ≥2 cardiovascular risk factors. However, ≥50% carotid stenosis was only observed in 11.0% of patients with less than 14 years interval after RT, 15.7% of patients with less than 2 cardiovascular risk factors, and 7.6% of patients with both a post-RT interval of less than 14 years and less than 2 cardiovascular risk factors.

## Discussion

The results of the present study showed that age and post-RT duration were significantly associated with most carotid atherosclerotic parameters, especially the carotid plaque burden. The findings are consistent with those reported in previous studies. Huang *et al.* documented that CIMT in post-RT NPC patients was significantly associated with age, post-RT duration and platelet count of the patients, and that age, post-RT duration and HbA1c level were significant predictors of carotid plaque [7]. In addition, Chang *et al.* found that age, radiation dose, post-RT duration and the presence of hyperlipidemia were associated with carotid plaque score in patients treated with neck RT for head and neck cancer [8]. Thus, age and post-RT duration are significant predictors of radiation-induced carotid atherosclerosis. In the present study, however, the cutoff values of age and post-RT duration for detecting carotid plaque were different from those in Huang's study. Age of 44.5 years and post-RT duration of 8.5 years were the cutoff values found in the present study, whereas the cutoff values of age and post-RT duration in Huang's study were 52.5 years and 42.5 months (3.5 years) respectively [7]. The discrepancy of the findings may attribute to the difference in post-RT duration of the patients and prevalence of carotid plaque between the present study and Huang's study. Since young patients (<45 years old) in the present study had even higher prevalence of carotid plaque (56.3% *vs* 36.2%) than patients in Huang's study (mean age of 52 years), and the mean post-RT duration of the patients in the present study was longer than that in Huang's study (12.5 *vs* 5 years), a lower cutoff value of age and a higher cutoff value of post-RT duration for detecting carotid plaque were found in the present study. In the present study, the two cutoff values, 44.5 years old and a post-RT interval of 8.5 years, were more powerful for predicting carotid plaque when used in combination. The highest prevalence of carotid plaque was shown in patients with both at least 45 years old and a post-RT interval of at least 9 years when compared to those with or without either one (*P_cor_*<0.001, 97.6% *vs* 79.5% *vs* 42.9%).

No previous study has investigated the predictors of radiation-induced ≥50% carotid stenosis. The present study found that post-RT duration was one of the significant predictors of ≥50% carotid stenosis in post-RT NPC patients and a post-RT interval of 13.5 years was the cutoff value for detecting the ≥50% stenosis. In addition, no previous study has reported the cumulative effects of cardiovascular risk factors on radiation-induced carotid atherosclerosis. The present study showed a significant association between the number cardiovascular risk factors and carotid plaque burden, indicating the cumulative effects of these factors on radiation-induced carotid atherosclerosis. The cutoff value of the number of cardiovascular risk factor was 1.5 for screening ≥50% carotid stenosis. Patients with ≥2 cardiovascular risk factors had greater carotid plaque score (*P_cor_*<0.05, 2.25±1.89 *vs* 1.19±1.09) and higher prevalence of ≥50% carotid stenosis (*P_cor_*<0.05, 40.7% *vs* 15.7%). It was more powerful to use the two cutoff values, a post-RT interval of 13.5 years and 1.5 cardiovascular risk factors, for screening ≥50% carotid stenosis. Highest risk of ≥50% carotid stenosis was found in patients with both a post-RT interval of at least 14 years and ≥2 cardiovascular risk factors when compared to those with or without either one (*P_cor_*<0.001, 63.6% *vs* 28.8% *vs* 7.8%).

Gender and hypertension only had association with CIMT and CAS respectively. However, NPC is common in men [1], and male sex did not associate with all carotid atherosclerotic parameters (especially carotid plaque burden) in post-RT duration. In addition, hypertension was only associated with CAS, and it was more powerful to use the number of cardiovascular risk factors for predicting radiation-induced carotid atherosclerosis. Therefore, the value of male sex and hypertension to be predictors of carotid atherosclerosis in post-RT NPC patients is limited. For the remaining potential predictors, no significant association was found with any of carotid atherosclerotic parameters. Thus, they may not be the predictors of carotid atherosclerosis in post-RT NPC patients.

## Limitations

Because radiation is a well-known risk of carotid atherosclerosis [17], the present study did not include subjects without RT as a control group. In addition, only 129 patients were recruited in the study. The small sample size limited the statistical power and thus there was a possibility that the association between some potential predictors and the radiation-induced carotid atherosclerosis in post-RT NPC patients may not be fully investigated. In addition, due to the small sample size, the present study only included nine potential predictors in the prediction models. Some other factors, such as medications, lifestyles, alcohol consuming and obesity, may also have underlying effects on radiation-induced carotid atherosclerosis. Thus, future studies are suggested to have a larger sample size and to investigate more potential predictors of radiation-induced carotid atherosclerosis.

## Conclusions

Age, post-RT duration and the number of cardiovascular risk factors are significant predictors of carotid atherosclerosis in post-RT NPC patients. In these patients, age of 44.5 years and post-RT duration of 8.5 years are the cutoff values for detecting carotid plaque, while post-RT duration of 13.5 years and 1.5 cardiovascular risk factors are the cutoff values for screening ≥50% carotid stenosis. The cutoff values used in combination are more powerful for predicting radiation-induced carotid plaque burden. Thus, NPC patients, who were aged 45 years or older, with post-RT duration of 9 years or above, and/or with ≥2 cardiovascular risk factors, should be more cautious of carotid atherosclerosis so that early diagnosis and timely treatment can be given to them.

## References

[1] Chang ET, Adami HO (2006) The enigmatic epidemiology of nasopharyngeal carcinoma. Cancer Epidemiology Biomarkers & Prevention 15:1765–1777. 10.1158/1055-9965.EPI-06-0353 17035381

[2] Glastonbury CM (2007) Nasopharyngeal carcinoma: the role of magnetic resonance imaging in diagnosis, staging, treatment, and follow-up. Top Magn Reson Imaging 18:225–235. 17893588 10.1097/RMR.0b013e3181572b3a

[3] Muzaffar K, Collins SL, Labropoulos N, Baker WH (2000) A prospective study of the effects of irradiation on the carotid artery. Laryngoscope 110:1811–1814. 11081590 10.1097/00005537-200011000-00007

[4] Lam WW, Leung SF, So NM, Wong KS, Liu KH, et al (2001) Incidence of carotid stenosis in nasopharyngeal carcinoma patients after radiotherapy. Cancer 92:2357–2363. 11745291 10.1002/1097-0142(20011101)92:9<2357::aid-cncr1583>3.0.co;2-k

[5] Lam WW, Yuen HY, Wong KS, Leung SF, Liu KH, et al (2001) Clinically underdetected asymptomatic and symptomatic carotid stenosis as a late complication of radiotherapy in Chinese nasopharyngeal carcinoma patients. Head Neck 23:780–784. 11505489 10.1002/hed.1111

[6] Li CS, Schminke U, Tan TY (2010) Extracranial carotid artery disease in nasopharyngeal carcinoma patients with post-irradiation ischemic stroke. Clin Neurol Neurosurg 112:682–686. 20579803 10.1016/j.clineuro.2010.05.007

[7] Huang TL, Hsu HC, Chen HC, Lin HC, Chien CY, et al (2013) Long-term effects on carotid intima-media thickness after radiotherapy in patients with nasopharyngeal carcinoma. Radiation Oncology 8. 10.1186/1748-717X-8-261 PMC3827874 24196030

[8] Chang YJ, Chang TC, Lee TH, Ryu SJ (2009) Predictors of carotid artery stenosis after radiotherapy for head and neck cancers. J Vasc Surg 50:280–285. 19631860 10.1016/j.jvs.2009.01.033

[9] Nadareishvili ZG, Rothwell PM, Beletsky V, Pagniello A, Norris JW (2002) Long-term risk of stroke and other vascular events in patients with asymptomatic carotid artery stenosis. Arch Neurol 59:1162–1166. 12117365 10.1001/archneur.59.7.1162

[10] Autret A, Pourcelot L, Saudeau D, Marchal C, Bertrand P, et al (1987) Stroke risk in patients with carotid stenosis. Lancet 1:888–890. 2882292 10.1016/s0140-6736(87)92861-3

[11] Alberti KG, Zimmet PZ (1998) Definition, diagnosis and classification of diabetes mellitus and its complications. Part 1: diagnosis and classification of diabetes mellitus provisional report of a WHO consultation. Diabet Med 15:539–553. 9686693 10.1002/(SICI)1096-9136(199807)15:7<539::AID-DIA668>3.0.CO;2-S

[12] Carretero OA, Oparil S (2000) Essential hypertension Part I: Definition and etiology. Circulation 101:329–335. 10645931 10.1161/01.cir.101.3.329

[13] Ford ES, Li CY, Pearson WS, Zhao GX, Mokdad AH (2010) Trends in hypercholesterolemia, treatment and control among United States adults. International Journal of Cardiology 140:226–235. 19081646 10.1016/j.ijcard.2008.11.033

[14] Yuan C, Lai CWK, Chan LWC, Chow M, Law HKW, et al (2014) Cumulative Effects of Hypertension, Dyslipidemia, and Chronic Kidney Disease on Carotid Atherosclerosis in Chinese Patients with Type 2 Diabetes Mellitus. Journal of Diabetes Research 10.1155/2014/179686 PMC4016922 24860832

[15] Matthews KA, Kuller LH, Sutton-Tyrrell K, Chang YF (2001) Changes in cardiovascular risk factors during the perimenopause and postmenopause and carotid artery atherosclerosis in healthy women. Stroke 32:1104–1111. 11340217 10.1161/01.str.32.5.1104

[16] Benjamini Y, Hochberg Y (1995) Controlling the False Discovery Rate - a Practical and Powerful Approach to Multiple Testing. Journal of the Royal Statistical Society Series B-Methodological 57:289–300.

[17] Gujral DM, Chahal N, Senior R, Harrington KJ, Nutting CM (2014) Radiation-induced carotid artery atherosclerosis. Radiother Oncol 110:31–38. 24044796 10.1016/j.radonc.2013.08.009

